# Negative impact of penicillin allergy labels on antibiotic use in hospitalized patients in Chinese Mainland

**DOI:** 10.1016/j.waojou.2022.100677

**Published:** 2022-08-24

**Authors:** Zihan Jiang, Hongting Zhang, Hao Xiao, Xiong Xiao, Juan Meng

**Affiliations:** a Department of Otorhinolaryngology, West China Hospital, Sichuan University, Chengdu, China; b West China School of Nursing, Sichuan University/Department of Otorhinolaryngology, West China Hospital, Sichuan University, Chengdu, China; c Allergy Center, West China Hospital, Sichuan University, Chengdu, China; d Department of Epidemiology and Biostatistics, West China School of Public Health and West China Fourth Hospital, Sichuan University, Chengdu, China

**Keywords:** Drug hypersensitivity, Antibiotic allergy, Penicillin allergy, Allergy label, Antimicrobial stewardship

## Abstract

**Background:**

Penicillin allergy labels have gained increasing global attention. However, to date, there are no data on the influence of penicillin allergy labels on patients in Chinese mainland.

**Methods:**

This retrospective study reviewed the electronic health record (EHR) of hospitalized patients between June 1, 2018 and May 31, 2019. Patients with a penicillin allergy record were included in the allergy group. Every allergy patient was matched with 4 control patients by using propensity score-based matching to make sure the following were balanced: age, sex, date of admission, and the main diagnosis. We estimated the prevalence of penicillin allergy labels and compared the antibiotic prescription patterns and other clinical outcomes between the 2 groups.

**Results:**

A total of 5691 patients and 22 585 patients were included in the allergy group and control group, respectively. The prevalence of penicillin allergy labels among the hospitalized patients in this study was 4.00%. Compared to the control group, significantly fewer patients in the allergy group were prescribed penicillins and most cephalosporins, while a larger proportion of allergy patients received clindamycin (10.02% vs 5.41%, p < 0.001) and some higher-class antibiotics, such as monobactams (1.81% vs 0.54%, p < 0.001), carbapenems (5.80% vs 4.98%, p = 0.014), macrolides (0.60% vs 0.25%, p < 0.001), and quinolones (17.62% vs 12.40%, p < 0.001). Allergy patients also had longer hospital stays and a greater need to consult infection specialists.

**Conclusion:**

The prevalence of penicillin allergy labels was 4.00% in Chinese hospitalized patients. Penicillin allergy labels could cause irrational antibiotic prescribing, prolonged hospital stays, and greater consultation needs.

## Introduction

Penicillins are one of the most common causes of drug allergies and fatal anaphylaxis.^1^^,^^2^ The prevalence of penicillin allergy labels ranges from 5% to 15% worldwide,^3^, ^4^, ^5^, ^6^, ^7^ yet only 2%–10% of subjects test positive for a penicillin allergy.^8^^,^^9^ Although there are many falsely labeled allergies and the ratio of genuine allergies is low, it is still a major concern while prescribing penicillin and treatment options.^10^ Due to insufficient knowledge of drug allergies, some clinicians accept the existing penicillin allergy labels or patients’ self-reported penicillin allergies without further verification. So, it is common for patients labeled as having a penicillin allergy to be given alternative antibiotics to avoid the risk of severe allergic reactions. Besides, clinicians tend to avoid prescribing other beta-lactam antibiotics, especially first- and second-generation cephalosporins, for fear of cross-reactivity. This often results in the unnecessary use of broad-spectrum and second-line antibiotics in patients with an unconfirmed penicillin allergy. Children and pregnant females are no exception.^11^^,^^12^ This subsequently leads to increasing surgical site infections,^13^ drug-resistant bacterial infections,^14^^,^^15^ and treatment failures.^16^ Some studies suggest that a documented penicillin allergy is a risk factor for higher medical costs^4^ and prolonged hospital stays.^14^ Moreover, for patients infected with COVID-19, penicillin allergy labels also impair COVID-19-related outcomes such as hospitalization, acute respiratory failure, intensive care unit (ICU) requirements, and mechanical ventilation.^17^ Therefore, removing false penicillin allergy labels is regarded as an important aspect of the antibiotic stewardship program.^18^^,^^19^

However, while penicillin allergy de-labelling has gained increasing global attention,^19^ there is still no report about the prevalence of penicillin allergy labels and its impact on patients in Chinese mainland where inaccurate penicillin allergy labels are suspected to be prevalent due to a lack of a standard penicillin allergy diagnosis algorithm. Thus, we conducted this study to investigate the prevalence of penicillin allergy labels in the inpatient electronic health record (EHR) and their impact on the antibiotic prescriptions and other clinical outcomes in Chinese hospitalized patients.

## Methods

### Study design

This retrospective study was conducted at West China Hospital, Sichuan University, Chengdu, China. The EHRs of hospitalized patients whose admission dates were between June 1, 2018 and May 31, 2019 were reviewed. Patients’ allergy history of drugs or food was asked and recorded descriptively in the “History of allergy” section of their EHRs. Patients recorded as having a penicillin allergy were included in the penicillin allergy group, regardless of their age or gender. To avoid considerable confounders, every patient in the penicillin allergy group was matched with up to 4 nearest control patients who had no penicillin allergy history through propensity score-based matching. The 4 factors included in the propensity score model were: gender (exact matching), age (within 12 months of each other), date of admission (within 1 month of each other), and the main diagnosis at discharge (exact matching to the first 3 characters of the ICD-10 codes). Any patient with a penicillin allergy who failed to be matched with at least 1 control subject was deleted from the analyses. If an allergy patient had 2 or more hospitalizations, the admission when he/she was first labeled with a penicillin allergy was adopted. For control patients, sampling without replacement was used. Patient characteristics, admission department, antibiotics used during hospitalization, length of hospital stays, and other medical information were collected. The antibiotics investigated in this study are shown in the Supplemental Appendix.

### Outcomes

In this study, we mainly focused on the prevalence of penicillin allergy labels and the differences in antibiotic use during hospitalization in patients with or without a penicillin allergy label including the proportions of patients using each antibiotic class or individual antibiotics and the time of medication. Only systemic use of antibiotics was considered including muscular, intravenous, and oral administration. Besides, the duration of hospital stays and the incidence of some clinical events, like resuscitation, ICU admission, consultation of infectious disease specialists, fungal infection, and drug-resistant bacterial infections, were also compared between the penicillin allergy group and the control group. Infections were identified according to the clinical bacteria culture results. Drug-resistant bacteria in this hospital included carbapenem-resistant *Acinetobacter baumannii* (CRAB), extended-spectrum beta-lactamase-producing bacteria (ESBLs), penicillin-resistant *Streptococcus pneumoniae* (PRSP), penicillin-intermediate resistant *Streptococcus pneumoniae* (PISP), *Clostridium difficile* (C.diff), carbapenem-resistant *Pseudomonas aeruginoa* (CRPA), carbapenem-resistant *Klebsiella pneumoniae* (CRKP), carbapenem-resistant *Enterobacteriaceae* (CRE), methicillin-resistant *Staphylococcus aureus* (MRSA), and vancomycin-resistant *Enterococci* (VRE).

### Statistical analysis

The intergroup differences were compared by using the *t*-test if the quantitative variables were normally distributed. Otherwise, the Wilcoxon rank-sum test was used. For categorical variables, the difference between groups was compared by using the Chi-squared test or the Fisher's exact test. A two-tailed *p*-value < 0.05 was considered to indicate statistical significance. Subgroup analyses were performed by sex and age group (under 18, between 18 and 64, and 65 or over). All analyses were performed in R (version 3.6.2, the R Foundation, Vienna, Austria).

## Results

### Characteristics of the study population

Among the 142 336 hospitalized patients during the year, 12 862 had documented drug allergies (9.04%). Penicillin allergy was the most common type of all, with 5691 patients having a penicillin allergy label (4.00%). This was followed by allergy labels of sulfonamides (2.37%), cephalosporins (1.30%), and quinolones (0.26%). Most of the allergy records lacked essential details of patients’ reactions to drugs, such as the date, severity, and manifestations of their reactions. All of the 5691 patients were matched with at least 1 unique control patient and 5584 (98.12%) of them were matched with 4 unique control patients. Overall, there were 22 585 unique control patients identified.

The characteristics of the patients in the allergy and control groups are shown in Table 1. All the study patients were Chinese. The median age was 55 years old, and the percentage of female patients was 58.94% in both groups. The main diagnoses of patients were divided into 17 categories based on ICD-10 coding. Patients with neoplasms accounted for the highest proportion (18.03% and 18.10% in the allergy and control groups respectively). Patients in the penicillin allergy group had slightly lower BMI than control patients.

**Table 1:** Overall characteristics of the study population.

	Allergy (n = 5691)	Control (n = 22,585)	p-value
Age^a^	55 (41, 69)	55 (42, 68)	0.484
Body mass index^a^	22.64 (20.07, 25.11)	22.74 (20.31, 25.11)	0.047
Female, n (%)	3354 (58.94)	13,312 (58.94)	1.000
Categories of main diagnoses based on ICD-10, n (%)			
Neoplasms	1026 (18.03)	4088 (18.10)	0.915
Diseases of digestive system	762 (13.39)	3036 (13.44)	0.934
Diseases of circulatory system	742 (13.04)	2961 (13.11)	0.902
Diseases of musculoskeletal system and connective tissue	633 (11.12)	2530 (11.20)	0.884
Diseases of respiratory system	486 (8.54)	1913 (8.47)	0.887
Diseases of genitourinary system	324 (5.69)	1290 (5.71)	0.983
Other conditions	312 (5.48)	1246 (5.52)	0.944
Infectious diseases	239 (4.20)	907 (4.02)	0.555
Metal and nervous disorders	218 (3.83)	871 (3.86)	0.958
Abnormal clinical and laboratory findings	173 (3.04)	684 (3.03)	0.999
Diseases of eye and ear	161 (2.83)	636 (2.82)	0.994
Diseases of skin and subcutaneous tissue	160 (2.81)	629 (2.79)	0.950
Injury, poisoning, and other external causes	159 (2.79)	625 (2.77)	0.949
Congenital abnormalities	134 (2.35)	533 (2.36)	1.000
Endocrine and metabolic diseases	130 (2.28)	511 (2.26)	0.961
Hematological diseases	29 (0.51)	113 (0.50)	1.000
Diseases of obstetrics	3 (0.05)	12 (0.05)	1.000

a Median (25th quartile, 75th quartile). ICD-10, international classification of diseases-10th revision

### Antibiotic treatment

**Fig. 1:**
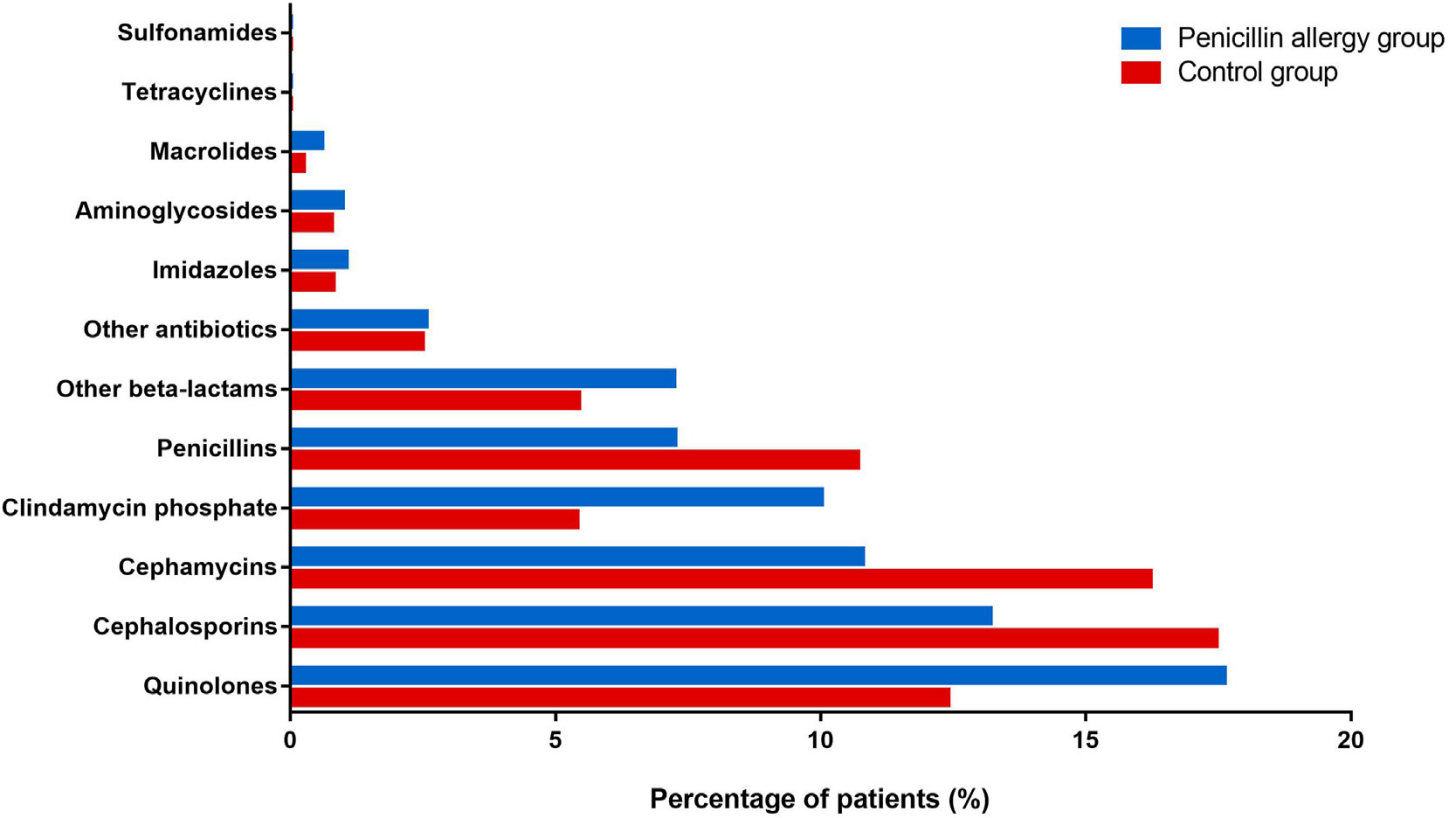
The consumption of each antibiotic class by the allergy and control group.

The percentages of patients who used antibiotics during hospitalization were similar in both groups. However, there were significant differences in the use of each antibiotic class (data is shown in Table 2 and Fig. 1). The detailed data for individual antibiotics is shown in Supplemental Table 1. Compared with 10.70% of patients in the control group, only 7.26% of patients with a penicillin allergy label were prescribed penicillins during their hospital stays (*p* < 0.001). The intergroup difference was mainly reflected in anti-pseudomonas penicillins (piperacillin sulbactam and piperacillin tazobactam) and benzylpenicillins (benzathine benzylpenicillin).

**Table 2:** Antibiotic use of patients during hospitalization.

	Allergy (n = 5691)	Control (n = 22,585)	*p*-value
Used antibiotics during hospitalizations, n (%)	2823 (49.60)	11,438 (50.64)	0.165
Average duration of antibiotic use, days^a^	5.47 ± 11.15	5.35 ± 10.96	0.479
Penicillins, n (%)			<0.001
No	5278 (92.74)	20,168 (89.30)	
Yes	413 (7.26)	2417 (10.70)	
Penicillinase-resistant penicillins	4 (0.07)	20 (0.09)	0.866
Ampicillins	31 (0.54)	149 (0.66)	0.378
Anti-pseudomonas penicillins	388 (6.82)	2260 (10.01)	<0.001
Benzylpenicillins	4 (0.07)	55 (0.24)	0.017
Cephalosporins, n (%)			<0.001
No	4940 (86.80)	18,642 (82.54)	
Yes	751 (13.20)	3943 (17.46)	
First-generation cephalosporins	301 (5.29)	1793 (7.94)	<0.001
Second-generation cephalosporins	197 (3.46)	1085 (4.80)	<0.001
Third-generation cephalosporins	294 (6.17)	1243 (5.50)	0.331
Cephamycins, n (%)			<0.001
No	5077 (89.21)	18,924 (83.79)	
Yes	614 (10.79)	3661 (16.21)	
Other β-lactams, n (%)			<0.001
No	5279 (92.76)	21,356 (94.56)	
Yes	412 (7.24)	1229 (5.44)	
Monobactams	103 (1.81)	123 (0.54)	<0.001
Carbapenems	330 (5.80)	1125 (4.98)	0.014
Aminoglycosides, n (%)			0.148
No	5635 (99.02)	22,409 (99.22)	
Yes	56 (0.98)	176 (0.78)	
Macrolides, n (%)			<0.001
No	5657 (99.40)	22,529 (99.75)	
Yes	34 (0.60)	56 (0.25)	
Quinolones, n (%)			<0.001
No	4688 (82.38)	19,784 (87.60)	
Yes	1003 (17.62)	2801 (12.40)	
Imidazoles, n (%)			0.096
No	5631 (98.95)	22,401 (99.19)	
Yes	60 (1.05)	184 (0.81)	
Tetracyclines, n (%)	0 (0.00)	0 (0.00)	1.000
Sulfonamides, n (%)	0 (0.00)	0 (0.00)	1.000
Other antibiotics, n (%)			<0.001
No	4975 (87.41)	20,799 (92.1)	
Yes	716 (12.58)	1786 (7.9)	
Clindamycin phosphate	570 (10.02)	1222 (5.41)	<0.001
Vancomycin	111 (1.95)	396 (1.75)	0.345
Norvancomycin	18 (0.32)	65 (0.29)	0.828
Linezolid	28 (0.49)	111 (0.49)	1.000
Tygacycline	42 (0.74)	190 (0.84)	0.491

a Mean ± standard deviation

There were also significantly fewer prescriptions of first- (cefazolin and cefathiamidine) and second-generation (cefuroxime and cefaclor) cephalosporins in the penicillin allergy group than in the control group. The same was true for the cephamycins (cefoxitin and cefmetazole). By contrast, for other kinds of beta-lactams, the allergy group received more monobactams (aztreonam, *p* < 0.001) and carbapenems (*p* = 0.014) than the control group.

For non-beta-lactams, the ratio of patients who received macrolides (erythromycin and azithromycin), quinolones (levofloxacin and moxifloxacin), metronidazole, and clindamycin phosphate was significantly higher in the allergy group than in the control group.

The results of the subgroup analyses by sex were similar to those of the general population, but there was a slightly different pattern in specific age groups for some antibiotics. For example, for the subpopulation who were under 18, the ratio of penicillins receivers in the allergy group (5.14%) was slightly lower than in the control group (5.67%), but the difference was not statistically significant (*p* = 0.826). However, the use of vancomycin was more common in the allergy group (5.14% vs 2.62%, *p* = 0.037), which was not seen in the general population and in patients who were over 18 years old. Moreover, the intergroup differences in monobactams (aztreonam), carbapenems (imipenem cilastatin and meropenem), macrolides (erythromycin and azithromycin), and clindamycin phosphate were consistent with but more notable than those of the general population. The detailed data of the subgroup analyses is shown in Supplemental Table 2-3.

### Other outcomes

The duration of medication for each antibiotic was also analyzed. Patients in the penicillin allergy group received cephalosporins for 5.31 ± 5.96 days on average, which was longer than 4.89 ± 5.16 days for the control group (*p* = 0.031). For quinolones, allergy patients also had longer medication time than control patients (7.48 ± 6.11 days vs 7.08 ± 6.57 days, *p* = 0.01). There was no statistical difference for other antibiotics (data not shown).

The incidences of fungal and drug-resistant bacteria infections during hospitalization were slightly higher in penicillin allergy patients (4.80% and 2.55%) than in control patients (4.51% and 2.41%), but there was no statistical difference (Table 3). The subgroup analyses showed similar results (data not shown).

**Table 3:** Fungal and drug-resistant bacteria infections during hospitalization.

	Allergy (n = 5691)	Control (n = 22,585)	p-value
Fungal infection, n (%)	273 (4.80)	1019 (4.51)	0.376
Drug-resistant bacteria infection, n (%)	145 (2.55)	544 (2.41)	0.585
CRAB	40 (0.70)	180 (0.80)	0.524
ESBL	83 (1.46)	283 (1.25)	0.246
PRSP or PISP	6 (0.11)	18 (0.08)	0.733
C. diff	1 (0.02)	6 (0.03)	1.000
CRPA	12 (0.21)	48 (0.21)	1.000
CRKP	14 (0.25)	41 (0.18)	0.413
CRE	0 (0.00)	9 (0.04)	0.276
MRSA	17 (0.30)	44 (0.19)	0.177
VRE	0 (0.00)	1 (0.00)	1.000

CRAB, cabapemne resistant Acinetobacter baumannii; ESBL, extended-spectrum beta-lactamase; PRSP, penicillin-resistant Streptococcus pneumoniae; PISP, penicillin-intermediate resistant Streptococcus pneumoniae; C.diff, *Clostridium difficile*; CRPA, carbapenem-resistant Pseudomonas aeruginoa; CRKP, carbapenem-resistant *Klebsiella pneumoniae*; CRE, carbapenem-resistant Enterobacteriaceae; MRSA, methicillin-resistant *Staphylococcus aureus*; VRE, vancomycin-resistant Enterococci

Table 4 shows that patients with a penicillin allergy label had a significantly longer hospital stay than patients in the control group (10.78 ± 10.13 days vs. 10.35 ± 9.77 days, *p* < 0.001). The same was true in the subgroups analyses for females, males, patients aged under 18 and patients aged 65 or over.

**Table 4:** Clinical events during hospitalization.

	Length of hospital stays, day^a^	p-value	Resuscitation, n (%)	p-value	Admitted to ICU, n (%)	p-value	Having infection consults, n (%)	p-value
General population								
Allergy	10.78 ± 10.13	<0.001	386 (6.78)	0.457	28 (0.49)	1.000	286 (4.53)	0.027
Control	10.35 ± 9.77		1598 (7.08)		110 (0.49)		979 (4.33)	
Female								
Allergy	10.29 ± 9.49	0.004	213 (6.35)	1.000	14 (0.42)	0.549	152 (4.53)	0.039
Control	9.97 ± 9.32		847 (6.36)		44 (0.33)		498 (3.74)	
Male								
Allergy	11.47 ± 10.96	0.041	173 (7.40)	0.285	14 (0.60)	0.654	134 (5.73)	0.316
Control	10.91 ± 10.35		751 (8.10)		66 (0.71)		481 (5.19)	
Age <18								
Allergy	9.62 ± 10.03	0.017	8 (2.57)	1.000	2 (0.64)	0.613	15 (4.82)	0.070
Control	8.14 ± 7.80		30 (2.54)		3 (0.25)		31 (2.62)	
18 ≤ Age<65								
Allergy	10.38 ± 8.16	0.051	237 (6.84)	0.872	14 (0.40)	1.000	178 (5.13)	0.037
Control	10.17 ± 8.38		969 (6.93)		58 (0.41)		601 (4.30)	
Age≥65								
Allergy	11.68 ± 12.94	0.017	141 (7.37)	0.336	12 (0.63)	1.000	93 (4.86)	0.777
Control	11.05 ± 12.13		599 (8.07)		49 (0.66)		347 (4.67)	

a Mean ± standard deviation

During hospitalization, 5.00% of patients in the allergy group accepted infection consultations, while only 4.30% of patients in the control group accepted (*p* = 0.027). Subgroup analyses showed that in females (*p* = 0.039) and patients aged between 18 and 64 (*p* = 0.037), the ratios of patients who accepted infection consultations were higher in the penicillin allergy group. No significant difference was found between the allergy and control groups for the ratios of resuscitation or ICU admission.

## Discussion

Despite the limited scope of a single center and the relatively small sample size, this study was the first epidemiological study on penicillin allergy labels in Chinese mainland. The prevalence of the penicillin allergy label in hospitalized patients was 4.00%. This is lower than the previously published data of western countries,^4^, ^5^, ^6^, ^7^ which is in accord with the findings of another study that claims Asian race is a protective factor against penicillin allergy.^3^ In Chinese mainland, there is a regulation that an intradermal test should be routinely performed before using penicillins. This is against the current consensus and may cause more false positive results.^20^ A small sample study of ours revealed that 44.56% of Chinese patients (291/653) had been labeled with penicillin allergy because of a previously positive result of the routine penicillin skin test (RPST), but only 1 out of 17 (5.89%) was confirmed to have a genuine penicillin allergy through the standard penicillin allergy diagnosis algorithm.^21^ Therefore, the prevalence of penicillin allergy labels in hospitalized patients may be lower if the routine screening requirement is cancelled.

The antibiotic medication during hospitalization was quite different between the allergy and control groups. Despite the same main diagnosis, fewer allergy patients received penicillins and cephalosporins compared with the controls. There were significant differences in anti-pseudomonas penicillins, benzylpenicillins, first- and second-generation cephalosporins and cephamycins. This indicates that some patients did not receive the first-line antibacterial treatment due to their penicillin allergy records. On the other hand, 7.26% of patients in the penicillin allergy group were still prescribed penicillins during their hospitalizations. It could be because these patients were labeled as penicillin allergy due to their previously positive results of RPST. However, in their current hospitalization, they passed the RPST and were administered penicillins uneventfully. This also reflects the high false positive rate of RPST in Chinese mainland. The decrease in the use of cephalosporins and cephamycins usually results from the concern of possible cross-reactivity between penicillins and cephalosporins because of their similar R1 side chain.^22^ In fact, in a population-based study, the rate of all new cephalosporins allergies in patients with and without a penicillin allergy record was only 1.13% and 0.39%, respectively. In addition, the rate of cephalosporins-associated anaphylaxis was rare (3 of 127,125 courses vs. 7 of 845,923 courses).^23^ In another study, prescribing more cephalosporins (especially first-generation cephalosporins) to patients with a penicillin allergy record did not cause additional incidence of anaphylaxis, new cephalosporin allergies, antibiotic treatment failure, all-cause mortality, hospitalization days, or new infections.^24^

Our data also showed an increased use of clindamycin, monobactams (aztreonam), carbapenems, macrolides (erythromycin and azithromycin), and quinolones (levofloxacin and moxifloxacin) in patients with a penicillin allergy label. According to the World Health Organization (WHO) AWaRe classification, carbapenems, macrolides, and quinolones are classified as Watch antibiotics and should be used for limited indications because they have a higher risk of resistance (particularly methicillin-resistant Staphylococcus aureus - MRSA and extended-spectrum beta-lactamase - ESBL), while aztreonam is one of the Reserve antibiotics which should be used when other antibiotics have failed or are not suitable.^25^ Carbapenems, clindamycin, and aztreonam are also considered risk factors for *C. difficile* infections.^26^^,^^27^ Accordingly, penicillin allergy labels proved to be an important contributor to inappropriate antibiotic use for Chinese patients. This is consistent with studies from other countries.^5^^,^^14^^,^^24^^,^^28^ In the current study, this influence on some Watch (imipenem cilastatin, meropenem, erythromycin, azithromycin, vancomycin) and Reserve (aztreonam) antibiotics was more notable in patients under 18. These alternatives were consumed more significantly by the penicillin allergy patients in the pediatric subgroup. It could be because the antibiotic options for children are limited. Carbapenems, monobactams and macrolides are safer alternatives for children in the context of penicillin allergy. This partly explains the findings of an international research study that showed pediatric patients in China had the lowest use of Access antibiotics (generally narrow-spectrum antibiotics) and the second highest use of Watch antibiotics across 56 countries.^29^

However, in the current study, the antibiotic prescription pattern for the control patients was also different from other countries. The data on antibiotic consumption in the European Union suggests that penicillins make up most of the antibiotic consumption. This is followed by cephalosporins and other beta-lactams, and quinolones.^30^ By contrast, our data showed cephamycins and cephalosporins were the most frequently used categories by control patients. The use of quinolones also exceeded the use of penicillins. And 8 of the top 10 antibiotics used by the control group were Watch antibiotics. This was undesirable in terms of antibiotic stewardship. It resulted from many factors, including the availability of antibiotics, doctors’ knowledge and preferences, and national guidelines.^31^ Therefore, measures should be taken to improve antibiotic prescriptions in China at every level, from personal to regulatory.

Unlike some studies conducted abroad,^14^^,^^15^ we failed to find any differences in the rates of fungal and drug-resistant bacterial infections between the allergy and control group. This could be attributed to the small sample size and inappropriate use of antibiotics in patients without an allergy label. For other clinical outcomes, we found that allergy patients had longer hospital stays and needed more consultations from infectious disease doctors. This confirms the additional medical cost cause by penicillin allergy labels and the financial benefits of de-labeling in patients with an unconfirmed record of penicillin allergy.^14^^,^^32^

One of the limitations of this study was that, to get as many samples as possible, only 4 conditions were adopted in the propensity-based matching. But there might be other potential confounders that influenced the results. For example, doctors' knowledge, experience, and preferences of antibiotics might differ. Patients’ comorbidities could also have influenced the treatment options despite having the same main diagnosis. Additionally, this single-center study cannot represent the overall status in Chinese mainland. Multi-center epidemiological data with a larger sample size is needed.

## Conclusion

The prevalence of penicillin allergy labels was 4.00% in the Chinese hospitalized population. The penicillin allergy label might cause irrational antibiotic prescriptions, prolonged hospital stays, and greater consultation needs. Given the adverse impact of penicillin allergy labels on individual and public health, it is urgent to promote the antibiotic stewardship program in Chinese mainland by standardizing the diagnosis algorithm for penicillin allergies, removing false allergy labels, and using antibiotics more reasonably.

## Abbreviations

ICU, intensive care unit; EHR, electronic health record; ICD-10, international classification of diseases-10th revision; CRAB, cabapemne resistant Acinetobacter baumannii; ESBL, extended-spectrum beta-lactamase; PRSP, penicillin-resistant Streptococcus pneumoniae.; PISP, penicillin-intermediate resistant Streptococcus pneumoniae.; C.diff, *Clostridium difficile*.; CRPA, carbapenem-resistant Pseudomonas aeruginoa; CRKP, carbapenem-resistant *Klebsiella pneumoniae*.; CRE, carbapenem-resistant Enterobacteriaceae; MRSA, methicillin-resistant *Staphylococcus aureus*; VRE, vancomycin-resistant Enterococci.

## Financial Support

We acknowledge the funding from: 1·3·5 project for disciplines of excellence–Clinical Research Incubation Project, 10.13039/501100013365West China Hospital, Sichuan University (2018HXFH026); Technology Innovation R&D Projects, Bureau of Science and Technology, Chengdu (2021-YF05-00473-SN); West China Nursing Discipline Development Special Fund Project, 10.13039/501100004912Sichuan University (HXHL21036).

## Availability of data and materials

We prefer not to publicly share the research data that may involve individual privacy. The data used or analyzed during the current study are available from the corresponding author on reasonable requests.

## Author contributions

Zihan Jiang and Hongting Zhang drafted the manuscript. Zihan Jiang and Hao Xiao collected the data. Xiong Xiao was responsible for the data cleaning and analysis. Juan Meng designed the study and revised the manuscript. All the authors reviewed the manuscript.

## Ethics approval

This study was approved by the Biomedical Research Ethics Committee of West China Hospital of Sichuan University, China (approval number: 2019 (123)) and registered on Chinese Clinical Trial Registry (registration number: ChiCTR1900021051).

## Authors’ consent for publication

All the authors have reviewed the final version of the manuscript and approve the submission.

## Declaration of competing interest

All authors report no competing interests.
