# Supplementary material for: Negative impact of penicillin allergy labels on antibiotic use in hospitalized patients in Chinese Mainland
Source: World Allergy Organ J. 2022 Aug 24;15(8):100677. doi: 10.1016/j.waojou.2022.100677 (PMC9428801; doi:10.1016/j.waojou.2022.100677)
Supplement: Multimedia component 1 [file mmc1.pdf]

Methods

Antibiotics prescribed in this hospital

Beta-lactam:

Penicillins:

Penicillinase-resistant penicillins: *oxacillin*

Ampicillins: *amoxicillin, amoxicillin clavulanate potassium, ampicillin*

Anti-pseudomonas penicillins: *piperacillin sulbactam sodium, piperacillin tazobactam sodium*

Benzylpenicillins: *penicillin G, benzathine benzylpenicillin*

Cephalosporins:

First-generation cephalosporins: *cefazolin, cefathiamidine*

Second-generation cephalosporins: *cefuroxime, cefaclor, cefprozil*

Third-generation cephalosporins: *cefixime, cefdinir, cefoperazone, ceftriaxone, ceftazidime, cefoperazone sulbactam sodium*

Fourth-generation cephalosporins: *cefepime*

Cephamycins: *cefoxitin, cefmetazole*

Other beta-lactams

Monobactams: *aztreonam*

Carbapenems: *imipenem cilastatin sodium, ertapenem, meropenem*

Aminoglycosides: *streptomycin, gentamicin, amikacin*

Macrolides: *erythromycin, clarithromycin, azithromycin*

Nitroimidazoles: *metronidazole, tinidazole*

Quinolones: *ciprofloxacin, levofloxacin, moxifloxacin*

Tetracyclines: *minocycline*

Sulfonamides: *sulfamethoxazole and trimethoprim*

Other antibiotics: *clindamycin phosphate, vancomycin, norvancomycin, linezolid, tygacycline*

Results

Supplemental Table 1. The use of specific antibiotics in penicillin allergy group and control group during hospitalization.

	Allergy (n=5691)	Control (n=22585)	*p*-value
Penicillins, n (%)			<0.001*
No	5278 (92.74)	20168 (89.30)	
Yes	413 (7.26)	2417 (10.70)	
Penicillinase-resistant penicillin (Oxacillin)	4 (0.07)	20 (0.09)	0.866
Ampicillins	31 (0.54)	149 (0.66)	0.378
Amoxicillin	13 (0.23)	66 (0.29)	0.500
Amoxicillin clavulanate potassium	18 (0.32)	83 (0.37)	0.650
Ampicillin	0 (0.00)	0 (0.00)	1.000
Anti-pseudomonas penicillins	388 (6.82)	2260 (10.01)	<0.001*
Piperacillin sulbactam sodium	206 (3.62)	1320 (5.84)	<0.001*
Piperacillin tazobactam sodium	193 (3.39)	1024 (4.53)	<0.001*
Benzylpenicillins	4 (0.07)	55 (0.24)	0.017*
Penicillin G	0 (0.00)	0 (0.00)	1.000
Benzathine benzylpenicillin	4 (0.07)	55 (0.24)	0.017*
Cephalosporins, n (%)			<0.001*
No	4940 (86.80)	18642 (82.54)	
Yes	751 (13.20)	3943 (17.46)	
First-generation cephalosporins	301 (5.29)	1793 (7.94)	<0.001*
Cefazolin	248 (4.36)	1427 (6.32)	<0.001*
Cefathiamidine	54 (0.95)	367 (1.62)	<0.001*
Second-generation cephalosporins	197 (3.46)	1085 (4.80)	<0.001*
Cefuroxime	125 (2.20)	668 (2.96)	<0.001*
Cefaclor	74 (1.30)	427 (1.89)	<0.001*
Cefprozil	0 (0.00)	2 (0.01)	1.000
Third-generation cephalosporins	294 (5.17)	1243 (5.50)	0.331
Cefixime	0 (0.00)	3 (0.01)	1.000
Cefdinir	0 (0.00)	0 (0.00)	1.000
Cefoperazone	0 (0.00)	0 (0.00)	1.000
Ceftriaxone	87 (1.53)	372 (1.65)	0.567

Ceftazidime	37 (0.65)	110 (0.49)	0.154
Cefoperazone sulbactam sodium	185 (3.25)	812 (3.60)	0.223
Fourth-generation cephalosporin (cefepime)	0 (0.00)	0 (0.00)	1.000
Cephamycins（cefoxitin, cefmetazole）			<0.001*
No	5279 (92.76)	21356 (94.56)	
Yes	614 (10.79)	3661 (16.21)	
Other β-lactams, n (%)			<0.001*
No	5279 (92.76)	21356 (94.56)	
Yes	412 (7.24)	1229 (5.44)	
Monobactams	103 (1.81)	123 (0.54)	<0.001*
Carbapenems	330 (5.80)	1125 (4.98)	0.014*
Imipenem cilastatin sodium	229 (4.02)	814 (3.60)	0.144
Ertapenem	0 (0.00)	0 (0.00)	1.000
Meropenem	119 (2.09)	392 (1.74)	0.081
Aminoglycosides, n (%)			0.148
No	5635 (99.02)	22409 (99.22)	
Yes	56 (0.98)	176 (0.78)	
Streptomycin	0 (0.00)	15 (0.07)	0.105
Gentamicin	7 (0.12)	23 (0.10)	0.833
Amikacin	49 (0.86)	139 (0.62)	0.052
Macrolides, n (%)			<0.001*
No	5657 (99.40)	22529 (99.75)	
Yes	34 (0.60)	56 (0.25)	
Erythromycin	16 (0.28)	32 (0.14)	0.035*
Clarithromycin	8 (0.14)	16 (0.07)	0.174
Azithromycin	10 (0.18)	8 (0.04)	0.001*
Quinolones, n (%)			<0.001*
No	4688 (82.38)	19784 (87.60)	
Yes	1003 (17.62)	2801 (12.40)	
Ciprofloxacin	102 (1.79)	325 (1.44)	0.058
Levofloxacin	423 (7.43)	1031 (4.56)	<0.001*
Moxifloxacin	536 (9.42)	1626 (7.20)	<0.001*
Imidazoles, n (%)			0.096

No	5631 (98.95)	22401 (99.19)	
Yes	60 (1.05)	184 (0.81)	
Metronidazole	44 (0.77)	120 (0.53)	0.040*
Tinidazole	19 (0.33)	74 (0.33)	1.000
Tetracyclines (minocycline), n (%)	0 (0.00)	0 (0.00)	1.000
Sulfonamides (Sulfamethoxazole and trimethoprim）, n (%)	0 (0.00)	0 (0.00)	1.000
Other antibiotics, n (%)			<0.001*
No	4975 (87.42)	20799 (92.09)	
Yes	716 (12.58)	1786 (7.91)	
Clindamycin phosphate	570 (10.02)	1222 (5.41)	<0.001*
Vancomycin	111 (1.95)	396 (1.75)	0.344
Norvancomycin	18 (0.32)	65 (0.29)	0.827
Linezolid	28 (0.49)	111 (0.49)	1.000
Tygacycline	42 (0.74)	190 (0.84)	0.490

Supplemental Table 2. The use of specific antibiotics by female and male patients during hospitalization.

	Female			Male		
	Allergy (n=3354)	Control (n=13312)	*p*-value	Allergy (n=2337)	Control (n=9273)	*p*-value
Ever used antibiotics during hospitalizations, n (%)	1570 (46.81)	6319 (47.47)	0.172	1253(53.62)	5119(55.20)	0.1754
Average duration of antibiotic use, day	4.85(10.66)	4.64(9.88)	0.278	6.35(11.77)	6.37(12.28)	0.940
Penicillins, n (%)			<0.001*			<0.001*
No	3152 (93.98)	12,124 (91.08)		2126 (90.97)	8044 (86.75)	
Yes	202 (6.02)	1188 (8.92)		211 (9.03)	1229 (13.25)	
Penicillinase-resistant penicillin (Oxacillin)	3 (0.09)	12 (0.09)	1.000	1 (0.04)	8 (0.09)	0.796
Ampicillins	16 (0.48)	79 (0.59)	0.502	15 (0.64)	70 (0.75)	0.662
Amoxicillin	5 (0.15)	32 (0.24)	0.424	8 (0.34)	34 (0.37)	1.000
Amoxicillin clavulanate potassium	11 (0.33)	47 (0.35)	0.955	7 (0.3)	36 (0.39)	0.660
Ampicillin	0 (0.00)	0 (0.00)	1.000	0 (0.00)	0 (0.00)	1.000
Anti-pseudomonas penicillins	189 (5.64)	1109 (8.33)	<0.001*	199 (8.52)	1151 (12.41)	<0.001*
Piperacillin sulbactam sodium	102 (3.04)	664 (4.99)	<0.001*	104 (4.45)	656 (7.07)	<0.001*
Piperacillin tazobactam sodium	91 (2.71)	479 (3.6)	0.014*	102 (4.36)	545 (5.88)	0.005*
Benzylpenicillins	2 (0.06)	24 (0.18)	0.181	2 (0.09)	31 (0.33)	0.072
Penicillin G	0 (0.00)	0 (0.00)	1.000	0 (0.00)	0 (0.00)	1.000
Benzathine benzylpenicillin	2 (0.06)	24 (0.18)	0.181	2 (0.09)	31 (0.33)	0.072
Cephalosporins, n (%)			<0.001*			<0.001*
No	2924 (87.18)	11016 (82.75)		2016 (86.26)	7626 (82.24)	
Yes	430 (12.82)	2296 (17.25)		321 (13.74)	1647 (17.76)	
First-generation cephalosporins	186 (5.55)	1096 (8.23)	<0.001*	115 (4.92)	697 (7.52)	<0.001*
Cefazolin	152 (4.53)	871 (6.54)	<0.001*	96 (4.11)	556 (6.00)	<0.001*
Cefathiamidine	35 (1.04)	225 (1.69)	<0.001*	19 (0.81)	142 (1.53)	0.011*
Second-generation cephalosporins	114 (3.4)	660 (4.96)	<0.001*	83 (3.55)	425 (4.58)	0.034*
Cefuroxime	79 (2.36)	425 (3.19)	0.013*	46 (1.97)	243 (2.62)	0.083
Cefaclor	36 (1.07)	240 (1.8)	<0.001*	38 (1.63)	187 (2.02)	0.254
Cefprozil	0 (0.00)	1 (0.01)	1.000	0 (0.00)	1 (0.01)	1.000
Third-generation cephalosporins	154 (4.59)	642 (4.82)	0.606	140 (5.99)	601 (6.48)	0.412
Cefixime	0 (0.00)	1 (0.01)	1.000	0 (0.00)	2 (0.02)	1.000
Cefdinir	0 (0.00)	0 (0.00)	1.000	0 (0.00)	0 (0.00)	1.000

Cefoperazone	0 (0.00)	0 (0.00)	1.000	0 (0.00)	0 (0.00)	1.000
Ceftriaxone	45 (1.34)	221 (1.66)	0.216	42 (1.8)	151 (1.63)	0.631
Ceftazidime	21 (0.63)	55 (0.41)	0.136	16 (0.68)	55 (0.59)	0.720
Cefoperazone sulbactam sodium	93 (2.77)	394 (2.96)	0.605	92 (3.94)	418 (4.51)	0.251
Fourth-generation cephalosporins (cefepime)	0 (0.00)	0 (0.00)	1.000	0 (0.00)	0 (0.00)	1.000
Cephamycins（cefoxitin, cefmetazole）			<0.001*			<0.001*
No	3030 (90.34)	11356 (85.31)		2047 (87.59)	7568 (81.61)	
Yes	324 (9.66)	1956 (14.69)		290 (12.41)	1705 (18.39)	
Other β-lactams, n (%)			<0.001*			0.002*
No	3146 (93.80)	12717 (95.53)		2133 (91.27)	8639 (93.16)	
Yes	208 (6.20)	595 (4.47)		204 (8.73)	634 (6.84)	
Monobactams	57 (1.70)	70 (0.53)	<0.001*	46 (1.97)	53 (0.57)	<0.001*
Carbapenems	163 (4.86)	538 (4.04)	0.039*	167 (7.15)	587 (6.33)	0.167
Imipenem cilastatin sodium	122 (3.64)	393 (2.95)	0.046*	107 (4.58)	421 (4.54)	0.981
Ertapenem	0 (0.00)	0 (0.00)	1.000	0 (0.00)	0 (0.00)	1.000
Meropenem	50 (1.49)	182 (1.37)	0.643	69 (2.95)	210 (2.26)	0.062
Aminoglycosides, n (%)			0.159			0.592
No	3325 (99.14)	13229 (99.38)		2310 (98.84)	9180 (99.00)	
Yes	29 (0.86)	83 (0.62)		27 (1.16)	93 (1.00)	
Streptomycin	0 (0.00)	6 (0.05)	0.471	0 (0.00)	9 (0.10)	0.275
Gentamicin	3 (0.09)	10 (0.08)	1.000	4 (0.17)	13 (0.14)	0.962
Amikacin	26 (0.78)	67 (0.50)	0.078	23 (0.98)	72 (0.78)	0.386
Macrolides, n (%)			0.003*			0.011*
No	3333 (99.37)	13276 (99.73)		2324 (99.44)	9253 (99.78)	
Yes	21 (0.63)	36 (0.27)		13 (0.56)	20 (0.22)	
Erythromycin	10 (0.30)	19 (0.14)	0.089	6 (0.26)	13 (0.14)	0.337
Clarithromycin	5 (0.15)	11 (0.08)	0.425	3 (0.13)	5 (0.05)	0.433
Azithromycin	6 (0.18)	6 (0.05)	0.026*	4 (0.17)	2 (0.02)	0.020*
Quinolones, n (%)			<0.001*			<0.001*
No	2800 (83.48)	11831 (88.87)		1888 (80.79)	7953 (85.77)	
Yes	554 (16.52)	1481 (11.13)		449 (19.21)	1320 (14.23)	
Ciprofloxacin	46 (1.37)	151 (1.13)	0.295	56 (2.40)	174 (1.88)	0.126
Levofloxacin	236 (7.04)	569 (4.27)	<0.001*	187 (8.00)	462 (4.98)	<0.001*

Moxifloxacin	310 (9.24)	857 (6.44)	<0.001*	226 (9.67)	769 (8.29)	0.037*
Imidazoles, n (%)			0.454			0.132
No	3325 (99.14)	13216 (99.28)		11491 (98.98)	2306 (98.67)	
Yes	29 (0.86)	96 (0.72)		119 (1.02)	31 (1.33)	
Metronidazole	20 (0.60)	59 (0.44)	0.311	24 (1.03)	61 (0.66)	0.083
Tinidazole	11 (0.33)	42 (0.32)	1.000	8 (0.34)	32 (0.35)	1.000
Tetracycline (minocycline) , n (%)	0 (0.00)	0 (0.00)	1.000	0 (0.00)	0 (0.00)	1.000
Sulfonamides (Sulfamethoxazole and trimethoprim), n (%)	0 (0.00)	0 (0.00)	1.000	0 (0.00)	0 (0.00)	1.000
Other antibiotics, n (%)			<0.001*			<0.001*
No	2949 (87.92)	12291 (92.33)		2026 (86.69)	8508 (91.75)	
Yes	405 (12.08)	1021 (7.67)		311 (13.31)	765 (8.25)	
Clindamycin phosphate	332 (9.90)	750 (5.63)	<0.001*	238 (10.18)	472 (5.09)	<0.001*
Vancomycin	53 (1.58)	188 (1.41)	0.517	58 (2.48)	208 (2.24)	0.541
Norvancomycin	8 (0.24)	34 (0.26)	1.000	10 (0.43)	31 (0.33)	0.627
Linezolid	13 (0.39)	53 (0.40)	1.000	15 (0.64)	58 (0.63)	1.000
Tygacycline	24 (0.72)	87 (0.65)	0.783	18 (0.77)	103 (1.11)	0.182

Supplemental Table 3. The use of specific antibiotics by different age groups during hospitalization.

	Age < 18			18≤Age < 65			Age≥65		
	Allergy (n=311)	Control (n=1182)	*p*-value	Allergy (n=3467)	Control (n=13980)	*p*-value	Allergy (n=1913)	Control (n=7423)	*p*-value
Ever used antibiotics during hospitalizations, n (%)	230 (73.95)	858 (72.59)	0.682	1706 (49.21)	6806 (48.68)	0.5944	887 (46.37)	3774 (50.84)	<0.001
Average duration of antibiotic use, day	7.35(12.52)	6.15(10.22)	0.080	5.27(10.71)	5.14(11.31)	0.553	5.52(11.66)	5.62(10.39)	0.722
Penicillins, n (%)			0.826			<0.001*			<0.001*
No	295 (94.86)	1115 (94.33)		3248 (93.68)	12765 (91.31)		1735 (90.70)	6288 (84.71)	
Yes	16 (5.14)	67 (5.67)		219 (6.32)	1215 (8.69)		178 (9.30)	1135 (15.29)	
Penicillinase-resistant penicillin (Oxacillin)	1 (0.32)	4 (0.34)	1.000	3 (0.09)	11 (0.08)	1.000	0 (0.00)	5 (0.07)	0.561
Ampicillins	0 (0.00)	5 (0.42)	0.550	25 (0.72)	100 (0.72)	1.000	6 (0.31)	44 (0.59)	0.1883
Amoxicillin	0 (0.00)	1 (0.08)	1.000	10 (0.29)	45 (0.32)	0.885	3 (0.16)	20 (0.27)	0.5305
Amoxicillin clavulanate potassium	0 (0.00)	4 (0.34)	0.586	15 (0.43)	55 (0.39)	0.860	3 (0.16)	24 (0.32)	0.3318
Ampicillin	0 (0.00)	0 (0.00)	1.000	0 (0.00)	0 (0.00)	1.000	0 (0.00)	0 (0.00)	1.000
Anti-pseudomonas penicillins	15 (4.82)	58 (4.91)	1.000	201 (5.80)	1114 (7.97)	<0.001*	172 (8.99)	1088 (14.66)	<0.001*
Piperacillin sulbactam sodium	7 (2.25)	22 (1.86)	0.832	103 (2.97)	635 (4.54)	<0.001*	96 (5.02)	663 (8.93)	<0.001*
Piperacillin tazobactam sodium	8 (2.57)	38 (3.21)	0.690	102 (2.94)	519 (3.71)	0.032*	83 (4.34)	467 (6.29)	<0.001*
Benzylpenicillins	0 (0.00)	2 (0.17)	1.000	2 (0.06)	34 (0.24)	0.051	2 (0.1)	19 (0.26)	0.329
Penicillin G	0 (0.00)	0 (0.00)	1.000	0 (0.00)	0 (0.00)	1.000	0 (0.00)	0 (0.00)	1.000
Benzathine benzylpenicillin	0 (0.00)	2 (0.17)	1.000	2 (0.06)	34 (0.24)	0.0517	2 (0.1)	19 (0.26)	0.329
Cephalosporins, n (%)			<0.001*			<0.001*			<0.001*
No	247 (79.42)	818 (69.2)		304 (87.71)	11736 (83.95)		1652 (86.36)	6088 (82.02)	
Yes	64 (20.58)	364 (30.8)		426 (12.29)	2244 (16.05)		261 (13.64)	1335 (17.98)	
First-generation cephalosporins	26 (8.36)	194 (16.41)	<0.001*	187 (5.39)	1101 (7.88)	<0.001*	88 (4.6)	498 (6.71)	<0.001*
Cefazolin	23 (7.4)	161 (13.62)	<0.001*	151 (4.36)	846 (6.05)	<0.001*	74 (3.87)	420 (5.66)	<0.001*
Cefathiamidine	4 (1.29)	33 (2.79)	0.189	36 (1.04)	256 (1.83)	<0.001*	14 (0.73)	78 (1.05)	0.259
Second-generation cephalosporins	20 (6.43)	130 (11.00)	0.023*	113 (3.26)	589 (4.21)	0.0121*	64 (3.35)	366 (4.93)	<0.001*
Cefuroxime	13 (4.18)	82 (6.94)	0.101	75 (2.16)	363 (2.6)	0.1617	37 (1.93)	223 (3)	0.014*
Cefaclor	8 (2.57)	48 (4.06)	0.288	39 (1.12)	232 (1.66)	0.0277	27 (1.41)	147 (1.98)	0.1221

Cefprozil	0 (0.00)	0 (0.00)	1.000	0 (0.00)	2 (0.01)	1.000	0 (0.00)	0 (0.00)	1.000
Third-generation cephalosporins	20 (6.43)	57 (4.82)	0.319	149 (4.3)	661 (4.73)	0.301	125 (6.53)	525 (7.07)	0.439
Cefixime	0 (0.00)	0 (0.00)	1.000	0 (0.00)	0 (0.00)	1.000	0 (0.00)	2 (0.03)	1.000
Cefdinir	0 (0.00)	0 (0.00)	1.000	0 (0.00)	0 (0.00)	1.000	0 (0.00)	0 (0.00)	1.000
Cefoperazone	0 (0.00)	0 (0.00)	1.000	0 (0.00)	0 (0.00)	1.000	0 (0.00)	0 (0.00)	1.000
Ceftriaxone	8 (2.57)	24 (2.03)	0.714	51 (1.47)	241 (1.72)	0.335	28 (1.46)	107 (1.44)	1.000
Ceftazidime	2 (0.64)	2 (0.17)	0.194	18 (0.52)	63 (0.45)	0.695	17 (0.89)	45 (0.61)	0.231
Cefoperazone sulbactam sodium	11 (3.54)	33 (2.79)	0.615	87 (2.51)	385 (2.75)	0.462	87 (4.55)	394 (5.31)	0.200
Fourth-generation cephalosporins (cefepime)	0 (0.00)	0 (0.00)	1.000	0 (0.00)	0 (0.00)	1.000	0 (0.00)	0 (0.00)	1.000
Cephamycins（cefoxitin, cefmetazole）			<0.001*			<0.001*			<0.001*
No	249 (80.06)	798 (67.51)		3100 (89.41)	11724 (83.86)		1728 (90.33)	6402 (86.25)	
Yes	62 (19.94)	384 (32.49)		367 (10.59)	2256 (16.14)		185 (9.67)	1021(13.75)	
Other β-lactams, n (%)			<0.001*			0.005*			0.013*
No	266 (85.53)	1120 (94.75)		3250 (93.74)	13275 (94.96)		1763 (92.16)	6961 (93.78)	
Yes	45 (14.47)	62 (5.25)		217 (6.26)	705 (5.04)		150 (7.84)	462 (6.22)	
Monobactam	21 (6.75)	14 (1.18)	<0.001*	57 (1.64)	69 (0.49)	<0.001*	25 (1.31)	40 (0.54)	0.001*
Carbapenems	27 (8.68)	51 (4.31)	0.003*	171 (4.93)	645 (4.61)	0.453	132 (6.90)	429 (5.78)	0.074
Imipenem cilastatin sodium	15 (4.82)	29 (2.45)	0.044*	123 (3.55)	484 (3.46)	0.257	91 (4.76)	301 (4.05)	0.193
Ertapenem	0 (0.00)	0 (0.00)	1.000	0 (0.00)	0 (0.00)	1.000	0 (0.00)	0 (0.00)	1.000
Meropenem	14 (4.50)	25 (2.12)	0.032*	57 (1.64)	207 (1.48)	0.530	48 (2.51)	160 (2.16)	0.397
Aminoglycosides, n (%)			0.887			0.349			0.154
No	309 (99.36)	1171 (99.07)		3430 (98.93)	13857 (99.12)		1896 (99.11)	7381 (99.43)	
Yes	2 (0.64)	11 (0.93)		37 (1.07)	123 (0.88)		17 (0.89)	42 (0.57)	
Streptomycin	0 (0.00)	0 (0.00)	1.000	0 (0.00)	14 (0.10)	0.126	0 (0.00)	1 (0.01)	1.000
Gentamicin	0 (0.00)	5 (0.42)	0.550	4 (0.12)	11 (0.08)	0.737	3 (0.16)	7 (0.09)	0.724
Amikacin	2 (0.64)	6 (0.51)	1.000	33 (0.95)	99 (0.71)	0.170	14 (0.73)	34 (0.46)	0.189
Macrolides, n (%)			<0.001*			0.005*			1.000
No	302 (97.11)	1178 (99.66)		3445 (99.37)	13939 (99.71)		1910 (99.84)	7412 (99.85)	

Yes	9 (2.89)	4 (0.34)		22 (0.63)	41 (0.29)		3 (0.16)	11 (0.15)	
Erythromycin	5 (1.61)	3 (0.25)	0.013*	10 (0.29)	26 (0.19)	0.327	1 (0.05)	3 (0.04)	1.000
Clarithromycin	0 (0.00)	1 (0.08)	1.000	8 (0.23)	11 (0.08)	0.032*	0 (0.00)	4 (0.05)	0.692
Azithromycin	4 (1.29)	0 (0.00)	0.001*	4 (0.12)	4 (0.03)	0.090	2 (0.10)	4 (0.05)	0.784
Quinolones, n (%)			0.100			<0.001*			<0.001*
No	306 (98.39)	1176 (99.49)		2821 (81.37)	12203 (87.29)		1561 (81.60)	6405 (86.29)	
Yes	5 (1.61)	6 (0.51)		646 (18.63)	1777 (12.71)		352 (18.40)	1018 (13.71)	
Ciprofloxacin	0 (0.00)	1 (0.08)	1.000	71 (2.05)	206 (1.47)	0.019*	31 (1.62)	118 (1.59)	1.000
Levofloxacin	3 (0.96)	5 (0.42)	0.467	298 (8.60)	688 (4.92)	<0.001*	122 (6.38)	338 (4.55)	0.001*
Moxifloxacin	2 (0.64)	0 (0.00)	0.059	317 (9.14)	1000 (7.15)	<0.001*	217 (11.34)	626 (8.43)	<0.001*
Imidazoles, n (%)			0.125			0.109			0.461
No	296 (95.18)	1148 (97.12)		3428 (98.88)	13865 (99.18)		1907 (99.69)	7388 (99.53)	
Yes	15 (4.82)	34 (2.88)		39 (1.12)	115 (0.82)		6 (0.31)	35 (0.47)	
Metronidazole	13 (4.18)	32 (2.71)	0.244	27 (0.78)	67 (0.48)	0.043*	4 (0.21)	21 (0.28)	0.757
Tinidazole	2 (0.64)	3 (0.25)	0.613	15 (0.43)	56 (0.40)	0.907	2 (0.10)	15 (0.20)	0.554
Tetracycline (minocycline) , n (%)	0 (0.00)	0 (0.00)	1.000	0 (0.00)	0 (0.00)	1.000	0 (0.00)	0 (0.00)	1.000
Sulfonamides (Sulfamethoxazole and trimethoprim), n (%)	0 (0.00)	0 (0.00)	1.000	0 (0.00)	0 (0.00)	1.000	0 (0.00)	0 (0.00)	1.000
Other antibiotics, n (%)			<0.001*			<0.001*			<0.001*
No	204 (65.59)	1000 (84.60)		3007 (86.73)	12771 (91.35)		1764 (92.21)	7028 (94.68)	
Yes	107 (34.41)	182 (15.40)		460 (13.27)	1209 (8.65)		149 (7.79)	395 (5.32)	
Clindamycin phosphate	94 (30.23)	151 (12.77)	<0.001*	370 (10.67)	840 (6.01)	<0.001*	106 (5.54)	231 (3.11)	<0.001*
Vancomycin	16 (5.14)	31 (2.62)	0.037*	72 (2.08)	289 (2.07)	1.000	23 (1.20)	76 (1.02)	0.579
Norvancomycin	1 (0.32)	4 (0.34)	1.000	14 (0.40)	50 (0.36)	0.806	3 (0.16)	11 (0.15)	1.000
Linezolid	3 (0.96)	6 (0.51)	0.607	17 (0.49)	65 (0.46)	0.955	8 (0.42)	40 (0.54)	0.632
Tygacycline	0 (0.00)	4 (0.34)	0.681	21 (0.61)	97 (0.69)	0.652	21 (1.10)	89 (1.20)	0.805
